# A Presurgical Unfavorable Prediction Scale of Endovascular Treatment for Acute Ischemic Stroke

**DOI:** 10.3389/fnagi.2022.942285

**Published:** 2022-06-30

**Authors:** Jingwei Li, Wencheng Zhu, Junshan Zhou, Wenwei Yun, Xiaobo Li, Qiaochu Guan, Weiping Lv, Yue Cheng, Huanyu Ni, Ziyi Xie, Mengyun Li, Lu Zhang, Yun Xu, Qingxiu Zhang

**Affiliations:** ^1^Department of Neurology of Drum Tower Hospital, Medical School and the State Key Laboratory of Pharmaceutical Biotechnology, Nanjing University, Nanjing, China; ^2^Institute of Brain Sciences, Nanjing University, Nanjing, China; ^3^Jiangsu Key Laboratory for Molecular Medicine, Medical School of Nanjing University, Nanjing, China; ^4^Jiangsu Province Stroke Center for Diagnosis and Therapy, Nanjing, China; ^5^Nanjing Neurology Clinic Medical Center, Nanjing, China; ^6^The Institute of Software, Chinese Academy of Sciences, Beijing, China; ^7^Department of Neurology, Nanjing First Hospital, Nanjing Medical University, Nanjing, China; ^8^Department of Neurology, Changzhou No.2 People's Hospital Affiliated to Nanjing Medical University, Changzhou, China; ^9^Department of Neurology, Northern Jiangsu People's Hospital, Clinical Medical School of Yangzhou University, Yangzhou, China; ^10^Department of Pharmacy of Drum Tower Hospital, Medical School, Nanjing University, Nanjing, China

**Keywords:** cerebral infarction, endovascular treatment, LightGBM algorithm, large-vessel occlusion, prediction model

## Abstract

**Objective:**

To develop a prognostic prediction model of endovascular treatment (EVT) for acute ischemic stroke (AIS) induced by large-vessel occlusion (LVO), this study applied machine learning classification model light gradient boosting machine (LightGBM) to construct a unique prediction model.

**Methods:**

A total of 973 patients were enrolled, primary outcome was assessed with modified Rankin scale (mRS) at 90 days, and favorable outcome was defined using mRS 0–2 scores. Besides, LightGBM algorithm and logistic regression (LR) were used to construct a prediction model. Then, a prediction scale was further established and verified by both internal data and other external data.

**Results:**

A total of 20 presurgical variables were analyzed using LR and LightGBM. The results of LightGBM algorithm indicated that the accuracy and precision of the prediction model were 73.77 and 73.16%, respectively. The area under the curve (AUC) was 0.824. Furthermore, the top 5 variables suggesting unfavorable outcomes were namely admitting blood glucose levels, age, onset to EVT time, onset to hospital time, and National Institutes of Health Stroke Scale (NIHSS) scores (importance = 130.9, 102.6, 96.5, 89.5 and 84.4, respectively). According to AUC, we established the key cutoff points and constructed prediction scale based on their respective weightings. Then, the established prediction scale was verified in raw and external data and the sensitivity was 80.4 and 83.5%, respectively. Finally, scores >3 demonstrated better accuracy in predicting unfavorable outcomes.

**Conclusion:**

Presurgical prediction scale is feasible and accurate in identifying unfavorable outcomes of AIS after EVT.

## Introduction

Cerebral infarction induced by acute large-vessel occlusion (LVO) in middle cerebral artery or vertebrobasilar artery has a high rate of disability and mortality (Goyal et al., 2016). In recent years, an increasing number of studies have found that endovascular treatment (EVT), which is used to perform recanalization in occluded large vessels, is an effective and plausible treatment that has further improved clinical outcome of patients (Lin et al., 2019; Jia et al., 2021). However, only about 30–40% patients achieved good outcomes even though intervention treatment was successful and blood flow was restored (Han et al., 2021; Jia et al., 2021). Therefore, it is an urgent task for us to explore a feasible prediction model for the outcomes of EVT of LVO.

According to recent clinical trials that have used new neuroimaging methods or biological markers, many risk factors would indicate poor outcomes for patients who received EVT for acute ischemic stroke (AIS) (Brugnara et al., 2020; Liu et al., 2021). To be specific, the risk factors, including brain edema, reperfusion injury, high National Institutes of Health Stroke Scale (NIHSS) scores, and blood-brain barrier damage were all associated with unfavorable prognosis (Chen et al., 2019; Heo et al., 2019; Brugnara et al., 2020; Butler et al., 2020; Liu et al., 2021). Even though research focused on prediction models using neuroimaging markers, biological markers, and neurological impairments is increasing, these predictors could not be used to make presurgical clinical decisions because they are examined and interpreted mostly during or after treatment.

Although logistic regression (LR) is widely applied in calculating disease-associated risk predictors, it still has limitations: it cannot provide satisfying accuracy, and it struggles with large numbers of variables (Dreiseitl and Ohno-Machado, 2002). Therefore, a prognosis model that predicts mid-term/short-term outcomes established by machine learning algorithm in stroke field began to gain attention (Heo et al., 2019; Brugnara et al., 2020; Liu et al., 2021). Furthermore, machine learning algorithm is able to deal with a huge number of complex variables and provides specific numerical values of different predictors (Deng et al., 2018; He et al., 2020; Castaneda-Vega et al., 2021). Among some widely used algorithms, light gradient boosting machine (LightGBM) is a classification model based on decision tree algorithm, with many advantages such as fast training speed, low memory consumption, high accuracy, and the ability to rapidly process massive data (Zhan et al., 2018; Chen et al., 2019; Shaker et al., 2021; Song et al., 2021; Liao et al., 2022).

In this study, 973 cases receiving EVT from four hospitals were enrolled and divided into favorable outcome and unfavorable outcome groups according to live independent ability. Machine learning model LightGBM was used to assess 20 related presurgical variables and construct prognostic prediction model to explore the major predictors for the first time. Afterward, the prediction scale was then established and further validated using raw data as well as new external data. The results suggested that prediction scoring mechanism would be a simple and pragmatic evaluation tool that can be widely used in clinical practice.

## Materials and Methods

### Data Collection and Processing

Data were retrospectively collected from patients who visited one of the four hospitals in Jiangsu Province in China from January 2018 to December 2020. The inclusion criteria were as follows: (1) age ≥18 years old; (2) within 24 h of onset; (3) neuroimaging-confirmed intracranial LVO induced AIS, including anterior cerebral artery (A1/A2), middle cerebral artery (M1/M2), basilar artery, intracranial internal carotid artery (T/L), vertebral artery (V4), and posterior cerebral artery (P1); (4) received EVT, including mechanical thrombectomy, angioplasty, intra-arterial thrombolysis, and stenting; and (5) with complete follow-up data with 90-day modified Rankin scale (mRS). The exclusion criteria were (1) malignant tumor; (2) incomplete data; (3) lost to follow-up; (4) severe heart, lung, and renal disease; and (5) pre-stroke mRS > 2 scores.

The external validation data, including 169 patients, was obtained from two of four hospitals from January to July 2021. The inclusion and exclusion criteria followed the above criteria as well.

Baseline data, including demographic characteristics, medical history, laboratory tests, clinical characteristics, occlusion locations, treatment details, and treatment outcomes, were collected for further analysis.

### Classification

The 973 patients from internal data were divided into favorable group (mRS ≤ 2 scores) and unfavorable group (mRS > 2 scores) based on 90-day mRS. A total of 20 important variables before EVT were selected to construct predictive model. These variables include general parameters, medical history, stroke etiology, treatments and key time points, laboratory test, and occlusion sites.

### Machine Learning Algorithms

We used a traditional statistical method and machine learning algorithms, namely, LR and LightGBM. LR is a classification model. It is widely applied in industrial issues and is simple to implement. However, it has limitations in accuracy because of overfitting and poor ability to handle too many variables. However, LightGBM can be trained quickly, costs low memory consumption, has high accuracy, and can support distributed and fast processing of massive data. More importantly, LightGBM was suitable for handling the structured data used in this study, which are of various types and some are missing. Other models need to perform null value processing, normalization, and other operations.

The LightGBM method involves the following steps: choice of a suitable dataset, selection of meaningful features, undersampling and splitting of dataset, training classification models, evaluation of classifiers' performance, and ranking of the weights of the influence factors.

Machine learning algorithm LightGBM was trained on the dataset consisting of 973 patients. The undersampling method was used to get balanced subsets. Then, the dataset was randomly split into two subsets, namely, training subset (80%) and test subset (20%). The training subset was used for establishing the model and the test subset for evaluating the model generalization on new data. This process was repeated 100 times to obtain average values. In this model, 20 important variables were trained as inputs to classify patients into favorable and unfavorable outcome groups. For the LightGBM model, parameters were set as follows, namely, number of estimators as 65, max depth of the tree as 6, and learning rate as 0.14. We tuned each parameter of the machine learning model and finally selected the optimal parameters. What needs illustration is that 20 presurgical variables are brought into our study based on their importance of influencing prognosis, including admitting blood glucose, age, time from onset to EVT, time from onset to hospital, NIHSS score, intravenous thrombolysis, hospital, stroke etiology, gender, transient ischemic attack (TIA), hypertension, atrial fibrillation, drinking, diabetes mellitus, smoking, time from hospital to puncture, occlusion site, coronary heart disease, hyperlipidemia, and stroke.

### Statistical Analysis

If baseline characteristics were in accordance with normal distribution, they were analyzed by one-way ANOVA. If not, the data were analyzed by Mann-Whitney *U*-test. LR with statistical analyses was performed with R (version 3.5.1, https://www.r-project.org/).

## Results

### Baseline Characteristics

**Figure 1:**
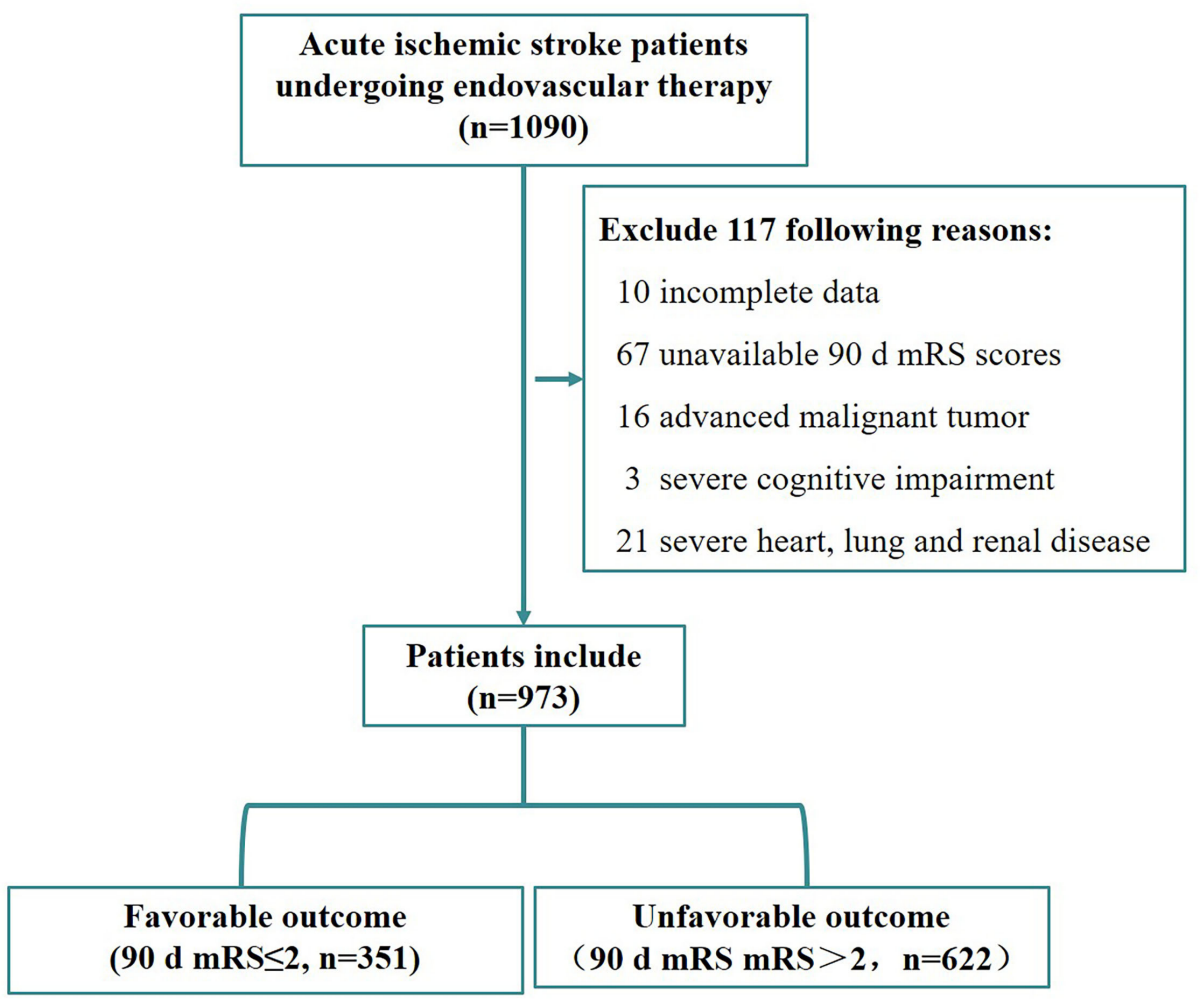
Flowchart of patient selection.

From January 2018 to December 2020, a total of 1,090 patients who underwent EVT for AIS were included in this study, and 117 patients were excluded due to certain reasons (Figure 1).

The baseline characteristics of favorable outcome group and unfavorable outcome group are summarized in Table 1, with the data presented as *n* (percent) or median [interquartile range (IQR)]. Patients in favorable outcome group were generally younger (65 vs. 73 years), consisted of more men (68.1 vs. 55.6%), had lower baseline NIHSS score (12 vs. 17), and had lower incidence of hypertension (63.2 vs. 70.4%), diabetes (16.8 vs. 23.2%), atrial fibrillation (26.8 vs. 35.5%), ischemic stroke (12.5 vs. 20.6%), and coronary heart disease (9.4 vs. 14.8%).

**Table 1:** Baseline characteristics of the patients in two groups.

**Characteristics**	**Postoperative mRS (0–2)**	**Postoperative mRS (3–6)**	***P* (Value)**
**Age, y, median (IQR)**	**65 (57–73)**	**73 (65–80)**	<0.001^***^
Male, *n* (%)	239 (68.1%)	346 (55.6%)	<0.001^***^
Female	112 (31.9%)	276 (44.4%)	
NIHSS score, median (IQR)	12 (8–16)	17 (12–25)	<0.001^***^
**Medical history**			
Hypertension, *n* (%)	222 (63.2%)	438 (70.4%)	0.021^*^
Diabetes, *n* (%)	59 (16.8%)	144 (23.2%)	0.019^*^
Dyslipidemia, *n* (%)	6 (1.7%)	13 (2.1%)	0.68
Atrial fibrillation, *n* (%)	94 (26.8%)	221 (35.5%)	0.005^**^
Ischemic stroke, *n* (%)	44 (12.5%)	128 (20.6%)	0.002^**^
TIA, *n* (%)	4 (1.1%)	8 (1.3%)	0.842
Smoking, *n* (%)	121 (34.5%)	147 (23.6%)	<0.001^***^
Drinking, *n* (%)	84 (23.9%)	89 (14.3%)	<0.001^***^
Coronary heart disease, *n* (%)	33 (9.4%)	92 (14.8%)	0.016^*^
**Stroke etiology**			
Large-artery atherosclerosis, *n* (%)	145 (41.3%)	231 (37.1%)	0.316
Cardioembolic, *n* (%)	155 (44.2%)	282 (45.3%)	
Tandem lesion, *n* (%)	51 (14.5%)	109 (17.5%)	
**Treatments and key time points**			
Intravenous thrombolysis, *n* (%)	106 (30.2%)	157 (25.2%)	0.094
Time from onset to hospital, min, median (IQR)	210 (120–360)	210 (120–315)	0.795
Time from onset to endovascular treatment, min, median (IQR)	310 (206–442)	300 (207–418)	0.487
Time from Door to Puncture, min, median (IQR)	80 (40–140)	85 (35–143)	0.894
**Laboratory test**			
Admitting blood glucose (mmol/L), median (IQR)	5.71 (4.97–6.755)	7.02 (5.702–8.908)	<0.001^***^
**Occlusion site**			
Anterior circulation	285 (81.2%)	482 (77.5%)	0.174
Posterior circulation	66 (18.8%)	140 (22.5%)	

*Favorable outcome group vs. unfavorable outcome group*,

* *p < 0.05*,

** *p < 0.01*,

*** *p < 0.001. IQR, interquartile range*;

*mRS, modified Rankin scale; NIHSS, national institutes of health stroke scale; TIA, transient ischemic attack*.

In addition, as shown in Supplementary Table 1, patients in favorable outcome group had higher successful revascularization rate (mTICI ≥ 2b, 93.2 vs. 82.8%), fewer mechanical thrombectomy times (2 [IQR, 1–2] vs. 2 [IQR, 1–3]), lower incidence of sICH (14.8 vs. 27.5%), shorter delay from door to reperfusion (151 vs. 180), shorter delay from puncture to reperfusion (60 vs. 85), etc. Compared to patients with unfavorable outcome, drinking and smoking rates were higher in favorable outcome group, which may be associated with a larger proportion of male patients. Furthermore, favorable outcome group showed lower levels of AST, admitting blood glucose, and creatinine.

### Clinical Prognostic of EVT for AIS Including Anterior and Posterior Circulation Infarction

**Figure 2:**
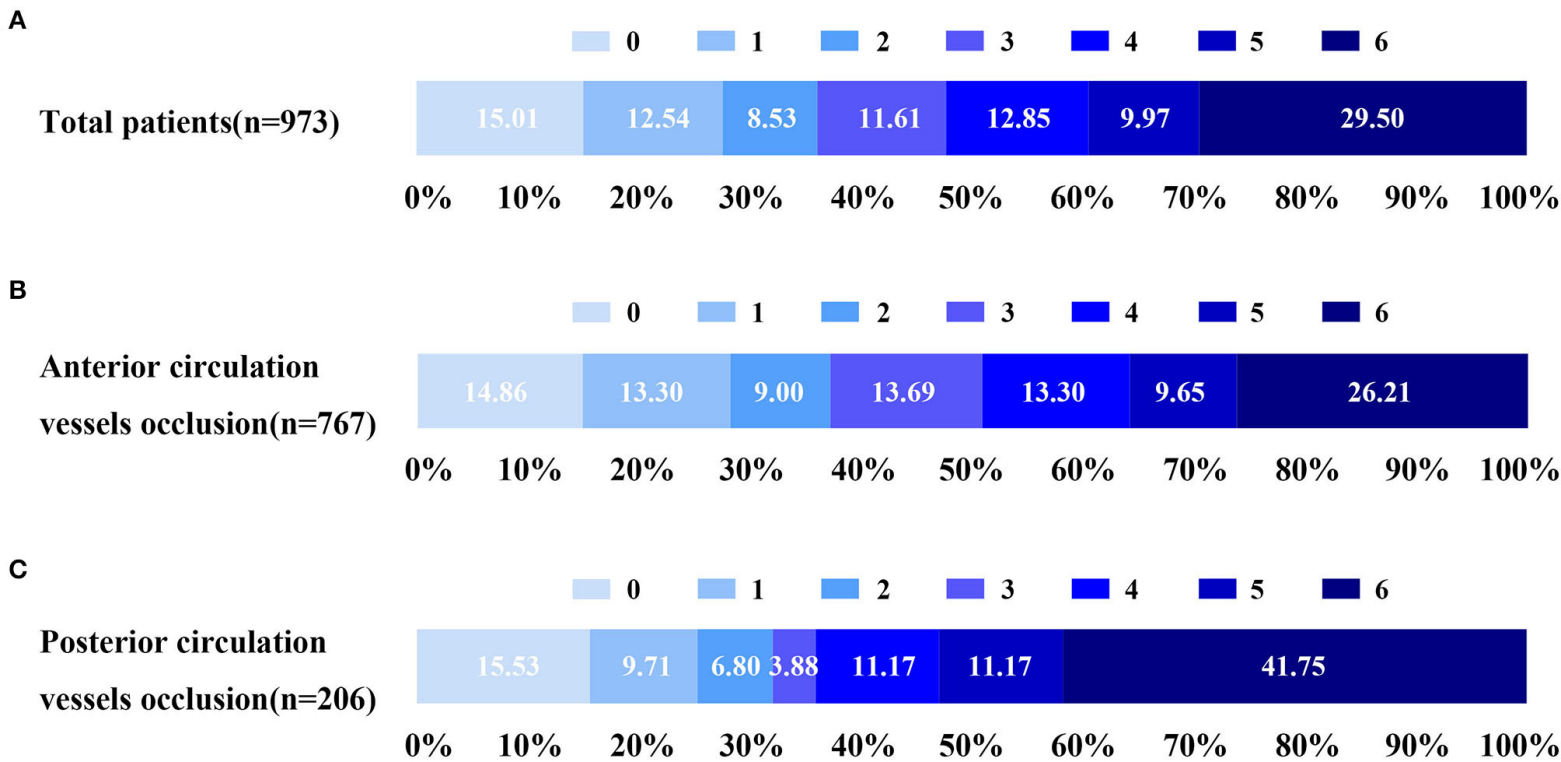
Distribution of 90-day mRS score of EVT for AIS in 973 patients from January 2018 to December 2020. **(A)** Distribution of mRS at 90 days for EVT. **(B)** Distribution of 90-day mRS for EVT at anterior circulation. **(C)** Distribution of 90-day mRS for EVT at posterior circulation. EVT, endovascular treatment; AIS, acute ischemic stroke; mRS, modified Rankin scale.

The primary outcome assessed by mRS is shown in Figure 2 and Supplementary Figure 1 for internal data and external data, respectively. Regarding internal data, favorable outcomes (mRS ≤ 2 scores) were achieved in 351/973 (36.07%) patients. In terms of the types of strokes, 767 patients (78.82%) underwent anterior circulation infarction (ACI) and 206 patients (21.18%) underwent posterior circulation infarction (POCI). A comparison of clinical outcomes between ACI and POCI showed that the patients who underwent EVT with ACI presented better clinical outcomes than POCI (37.16% in ACI vs. 32.04% in POCI) (Figure 2). Furthermore, the mortality in ACI group (201/767, 26.21%) was significantly lower than that in POCI group (86/206, 41.75%).

In contrast, the external data shown in Supplementary Figure 1 demonstrated that the percentage of favorable outcome was 72/169 (42.60%) and that of death was 38/169 (22.49%).

### Important Predictors for Long-Term Outcome of EVT for AIS

**Figure 3:**
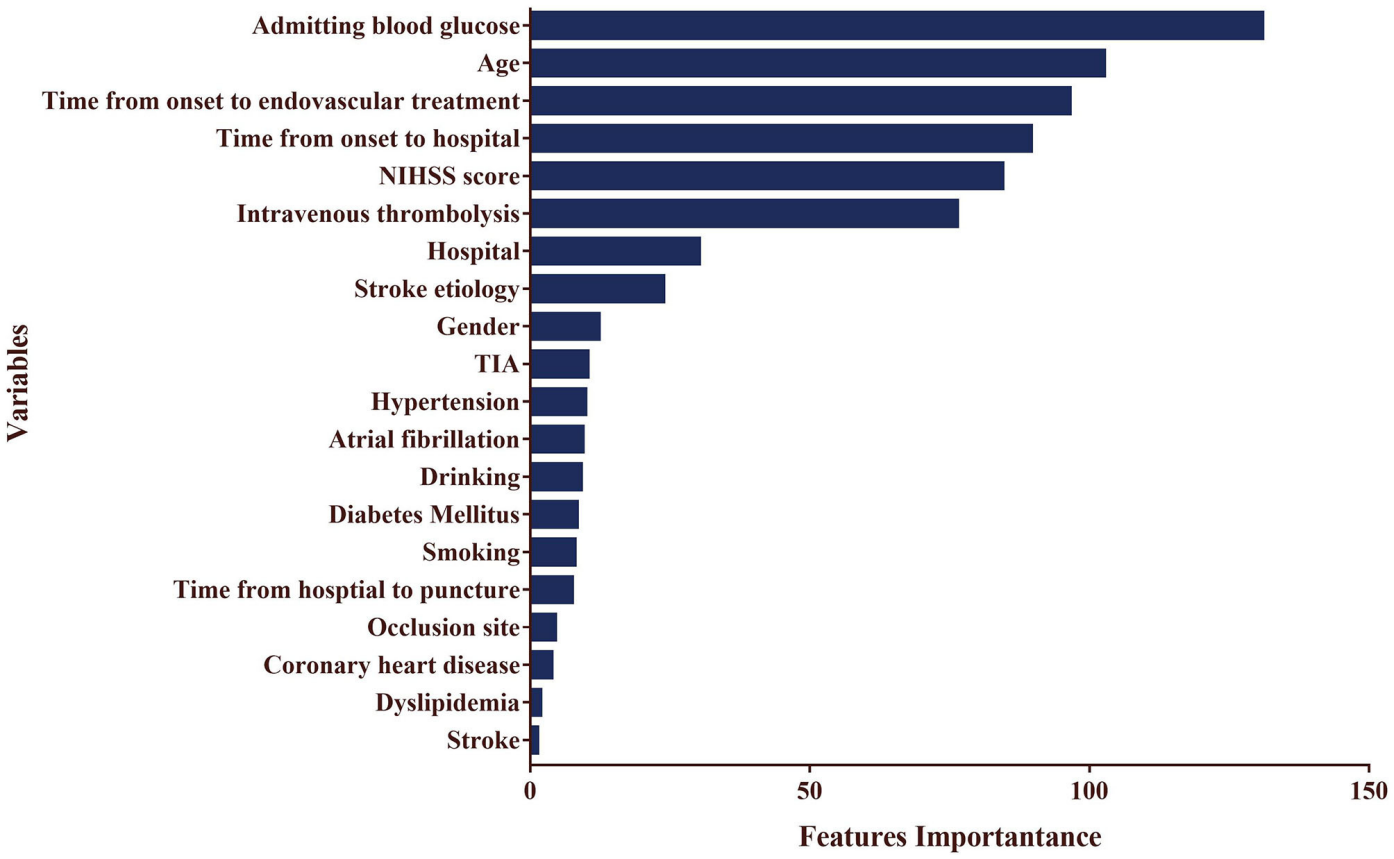
The weighting of outcome after EVT for AIS based on LightGBM model.

To establish an accurate presurgical prediction model for EVT, we collected as many variables as possible to increase the dimensions so as to avoid overfitting. Altogether 20 closely related parameters before EVT were selected. After training and testing the datasets 100 times, the LightGBM algorithm assigned different weightings to variables based on their significance. The top 5 important parameters for predicting outcomes of EVT for AIS were admitting blood glucose levels, age, onset to EVT time, onset to hospital time, and NIHSS scores (importance = 130.9, 102.6, 96.5, 89.5, and 84.4, respectively) (Figure 3).

In contrast, LR analysis showed that the following variables were significantly associated with unfavorable outcomes, namely, advanced age (beta = 0.052, *p* < 0.001), high baseline NIHSS score (beta = 0.097, *p* < 0.001), and high levels of admitting blood glucose (beta = 0.252, *p* < 0.001) (Table 2).

**Table 2:** Logistic regression analysis of the factors affecting the outcome of EVT for AIS.

**Variables**	**Beta**	**SE**	***P*(Value)**
Age, y	0.052	0.007	6.05*10^−14***^
Admitting blood glucose	0.252	0.037	1.45*10^−11***^
Baseline NIHSS Scores	0.097	0.011	2.00*10^−16***^

Favorable outcome group vs. unfavorable outcome group, ^***^p < 0.001.

**Figure 4:**
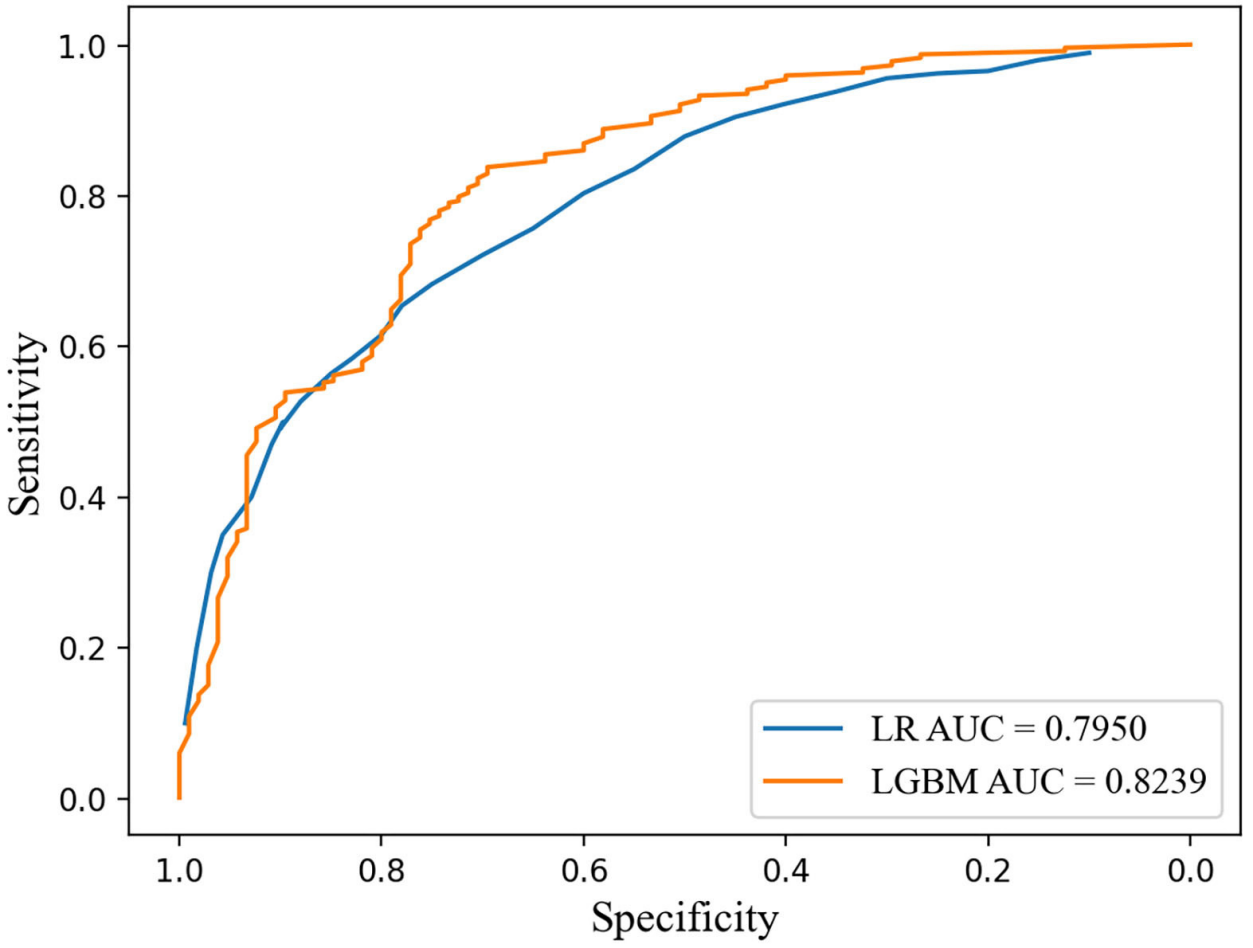
ROC curves of LightGBM model and logistic regression model for predicting unfavorable outcome after EVT for AIS. LightGBM, light gradient boosting machine; ROC, receiver operating characteristic.

Furthermore, we compared the two prediction models and found that LightGBM model performed better than LR model (0.738 vs. 0.701 in accuracy and 0.824 vs. 0.795 in area under the curve) (Supplementary Table 2; Figure 4). LightGBM method is thus suggested to be an accurate and feasible prediction model for unfavorable outcomes of EVT for AIS.

### Prediction Scale Might Be an Efficient Tool to Suggest Possible Outcomes of EVT for AIS

To further clarify the validity of important parameters found in LightGBM model in predicting outcomes, we first confirmed critical values and cutoff points using receiver operating characteristic (ROC) curve. The cutoff points of top 5 predictors for the prognosis were as follows: age was 67 years, NIHSS scores were 14, admitting blood glucose was 6.47 mmol/L, onset to EVT time was 380 min, and onset to hospital time was 178 min. In terms of age, we classified patients into two different groups and found that 51.5 and 25.6% patients achieved favorable outcome in ≤67 years age group and >67 years age group [odds ratio (OR), 3.08; 95% confidence interval (CI), 2.35–4.04], respectively (Supplementary Table 3). In contrast, we also divided patients into two different groups based on baseline NIHSS score. The results indicated that 52.7 and 21.4% patients gained independence in life in ≤14 scores group and >14 scores group (OR, 4.09; 95% CI, 3.10–5.41), respectively (Supplementary Table 4).

Therefore, we further established a prediction scale based on both the cutoff points of these predictors and their weighting values (Table 3). The components, scoring criteria, and assigned scores of the prediction scale were as follows: age > 67 years (= 2, if not = 0), NIHSS scores > 14 (= 2, if not = 1), admitting blood glucose > 6.47 mmol/L (= 1, if not = 0), onset to EVT time > 380 min (= 1, if not = 0), and onset to hospital time > 178 min (= 1, if not = 0). The scale was further validated using 973 internal patients' data, and we found that the sensitivity was 80.4% and area under the ROC curve was 0.72 (Table 4; Supplementary Figure 2). Additionally, we used another 169 external patients' data collected in 2021 to verify the prediction scale, and the resulting sensitivity was 83.5% (Table 4). Consequently, a score of 3 was identified as the cutoff point, and we found that prediction scale >3 presented higher accuracy when forecasting unfavorable outcomes at 76.7% (Supplementary Table 5). These results suggested that the prediction scale of EVT for AIS will help estimate the proportion of unfavorable prognosis.

**Table 3:** Prediction scores of EVT in AIS.

**Component**	**Scoring criteria**	**Scores**
Age, y	≤67	0
	>67	2
NIHSS scores	≤14	1
	>14	2
Admitting blood glucose, mmol/L	≤6.47	0
	>6.47	1
Onset to hospital time, min	≤178	0
	>178	1
Onset to EVT time, min	≤380	0
	>380	1

*The scores were assigned based on the weighting and clinical research*.

**Table 4:** The prognostic impact of prediction scale in EVT for AIS validated by internal and external data.

**Different data**	**Sensitivity [%]**	**Specificity [%]**	**AUC**
Raw data	80.4	56.7	0.723
External data	83.5	41.7	0.685

*Raw data, 973 patients from January 2018 to December 2020; external data, 169 patients from January to July in 2021*.

## Discussion

In this study, we used machine learning LightGBM model to analyze 20 important presurgical variables and constructed a prognostic model of EVT for LVO in real world for the first time and identified several key predictors based on the weightings of multidimensional features, including age, NIHSS scores, admitting blood glucose, onset to hospital time, and onset to EVT time. On this basis, we further established a prediction scale to assess the proportion of patients with unfavorable outcomes and confirmed that score of 3 was the key cutoff point that can distinguish unfavorable outcomes from favorable outcomes.

In recent years, several studies have revealed that brain edema, high mean blood pressure, greater blood pressure variability, and high NIHSS scores were associated with adverse outcome of successful embolectomy of AIS (Chen et al., 2019; Heo et al., 2019; Quinn and Drozdowska, 2019; Butler et al., 2020). In addition, Brugnara et al. (2020) utilized machine learning method to analyze pre- and postinterventional characteristics and found that premorbid mRS and final infarction volume were important predictors for 90-day mRS. However, their study has limitations as they were single-centered and their sample size was small, thus possibly resulting in the overfitting of machine learning algorithms.

In contrast, application of machine learning in disease prediction is widely accepted due to its advantages in processing massive and multidimensional data (Deng et al., 2018; Kamel et al., 2020; Castaneda-Vega et al., 2021). However, although many prediction models have been developed, few have been applied and proved efficient in clinical practice, especially in guiding clinical decision-making (Quinn and Drozdowska, 2019; Kaesmacher et al., 2020; Crowe et al., 2021; Zhu et al., 2021). By contrast, in this study, we incorporated large samples from four clinical centers, and the data were analyzed from many dimensions, including clinical variables, process variables, and biomarkers, to establish a prediction model that predicts long-term outcomes of EVT for AIS. Nevertheless, machine learning has limitations at the interpretation of the results. To avoid “black box” phenomenon of machine learning, we further applied traditional statistical method to analyze the data with the same variables.

In recent years, it is well recognized that age, stroke severity (NIHSS score or infarction volume), and treatment method (intravenous thrombolysis or arterial mechanical thrombectomy) are vital prognostic markers (Drozdowska et al., 2019; Quinn and Drozdowska, 2019; Crowe et al., 2021). In this study, we provided supporting evidence for a conclusion drawn from previous studies that patients younger than 67 years old and/or NIHSS score below 14 are more likely to achieve positive clinical outcome. These results can facilitate neurologists and patients to make treatment decisions.

In addition, as is consistent with our results, the delay from hospital arrival to puncture or from onset to reperfusion would worsen the outcome of mechanical thrombectomy for acute stroke (Bourcier et al., 2019; Kaesmacher et al., 2020; Snyder et al., 2020; Zhu et al., 2021). In this study, the model demonstrated the top 5 important variables, two of which are related to time, namely, time from onset to hospital, and time from onset to EVT. These results provide important supporting arguments for reducing the intervals of several time points and improving stroke quality control indicators in stroke centers in the future.

Moreover, the results of several randomized controlled studies were controversial on the effect of EVT with or without intravenous alteplase (De Marchis et al., 2019; Suzuki et al., 2021). The results of one-way ANOVA suggested that EVT plus intravenous alteplase was better at restoring functional independence of patients, but the difference was not statistically significant. However, the weighting of intravenous thrombolysis in the prognostic model should be prioritized, even though it needs further clinical trials for validation.

In this study, we applied classification model LightGBM method to predict the prognosis after EVT for AIS. In contrast to traditional LR, LightGBM model showed improved accuracy of prediction. What is worth noting is that established prediction scale based on major predictors might give us an opportunity to help patients and doctors make clinical decisions on the basis of this model.

### Strengths and Limitations

Our study has several limitations. First, the data collected from four hospitals were retrospective, which means that data were sometimes incomplete. The sample sizes can still be increased, especially the data in 2021. Secondly, the selected four hospitals were limited to Jiangsu Province and can be expanded to the eastern part of China. Thirdly, the prediction scale focused only on the presurgical variables, which means that the features during or after EVT were overlooked and therefore the specificity of the scoring system was limited. Finally, the lack of external data also limits the accuracy of data validation.

Nevertheless, there are several strengths in this study. The data obtained from four national stroke centers provided huge sample sizes and multiple dimensions. Combining machine learning with traditional statistical methods, we developed better prediction model that can avoid the typical shortcoming of machine learning model: “black box.” Furthermore, we established a predictive evaluation system – a prediction scale, validated its accuracy in internal and external data, and identified the cutoff points that can distinguish the higher and lower percentages of positive prognosis.

## Conclusion

This study demonstrated that the prediction model of EVT for AIS with machine learning algorithm LightGBM model was in general accurate and could be applied to clinical decision-making for LVO. Likewise, the prediction scale established based on the abovementioned model might be an accurate and feasible tool. Two risk factors were identified for unfavorable outcomes of EVT for AIS, namely, age > 67 years and NIHSS score > 14. Better outcomes of EVT could be achieved by minimizing the time intervals of stroke quality control key points and improving the techniques and devices of acute EVT.

## Data Availability Statement

The raw data supporting the conclusions of this article will be made available by the authors, without undue reservation.

## Ethics Statement

The studies involving human participants were reviewed and approved by Medical Ethics Committee of Nanjing Drum Tower Hospital. Written informed consent for participation was not required for this study in accordance with the national legislation and the institutional requirements.

## Author Contributions

QZ and YX designed the study. QZ and LZ wrote the first draft. JL, JZ, WY, and XL performed the EVT operations and quality control for the study. WZ, YC, HN, and QZ performed the statistical analysis. QG, WL, ZX, and ML collected the data. All authors contributed to the further drafts, read, and approved the final manuscript.

## Funding

This work was supported by the Key Research and Development Program of Jiangsu Province of China (BE2020620 to Dr. YX) and the National Natural Science Foundation of China (Nos. 82071304 and 81671149 to QZ).

## Conflict of Interest

The authors declare that the research was conducted in the absence of any commercial or financial relationships that could be construed as a potential conflict of interest.

## Publisher's Note

All claims expressed in this article are solely those of the authors and do not necessarily represent those of their affiliated organizations, or those of the publisher, the editors and the reviewers. Any product that may be evaluated in this article, or claim that may be made by its manufacturer, is not guaranteed or endorsed by the publisher.
