# Supplementary material for: A Presurgical Unfavorable Prediction Scale of Endovascular Treatment for Acute Ischemic Stroke
Source: Front Aging Neurosci. 2022 Jun 30;14:942285. doi: 10.3389/fnagi.2022.942285 (PMC9284674; doi:10.3389/fnagi.2022.942285)

Supplementary Marterials

Supplementary Table 1. Summary of treatments, secondary end point events and security indicator events. sICH, Symptomatic Intracranial Hemorrhage; mTICI, modified Treatment in Cerebral Infarction score; ALT, Alanine transaminase; AST, Aspartate aminotransferase. Favorable outcome group versus unfavorable outcome group, *P<0.05, **P<0.01, ***P<0.001.

Supplementary Table 2. The summary of evaluation metrics for each algorithm.

Supplementary Table 3. The effect of age on long-term outcome in EVT for AIS. Different age groups comparison (> 67 y group VS ≤67 group), $^{***}P$<0.001.

Supplementary Table 4. The effect of NIHSS score on long-term outcome in EVT for AIS. Different NIHSS scores groups comparison (> 14 scores group VS ≤14 scores group), ***P<0.001.

Supplementary Table 5. Effect of different score segments in prediction scale on prognosis of EVT for AIS. Different prediction scale scores groups comparison (> 3 scores group VS ≤3 scores group), $^{***}P$<0.001.

Supplementary Figure 1. Distribution of 90d mRS of EVT for AIS in 169 patients in 2021.

Supplementary Figure 2. ROC curve of the prediction scale for predicting outcomes after EVT for AIS in 973 patients from January 2018 to December 2020.

Supplementary Table 1. Summary of treatments, secondary end point events and security indicator events.

	postoperative mRS(0-2)	postoperative mRS (3-6)	P (Value)
**Treatments**			
Number of mechanical thrombectomy, n, median (IQR)	2.0 (1.0-2.0)	2.0 (1.0-3.0)	<0.001***
Local anesthesia, n (%)	338(96.3%)	570(91.6%)	0.005**
General anesthesia, n (%)	13(3.7%)	52(8.4%)	
Time from Puncture to reperfusion, min, median (IQR)	60.0 (40.0-90.75)	85.0 (55.0-120.0)	<0.001***
Time from onset to reperfusion, min, median (IQR)	392.0 (285.0-533.8)	405.0 (301.2-540.0)	0.416
**Operation mode**			
Aspiration, n (%)	9(2.6%)	13(2.1%)	0.493
Stent retriever only, n (%)	56(16.0%)	95(15.3%)	
Stent retriever and aspiration, n (%)	262(74.6%)	485(78.0%)	
Balloon dilatation, n (%)	12(3.4%)	18(2.9%)	
Others, n (%)	12(3.4%)	18(1.8%)	
**Blood pressure parameters**			
Intraoperative mean arterial pressure (mmHg), median (IQR)	101.0 (93.0-110.0)	102.0 (93.0-110.8)	0.482
Mean arterial pressure within 24 hours (mmHg), median (IQR)	92.0 (83.0-99.0)	94.0 (86.0-102.0)	<0.001***
**Surgical complications**			
sICH, n (%)	52(14.8%)	171(27.5%)	<0.001***
Contrast medium leakage, n (%)	51(14.5%)	116(18.6%)	0.102
Reperfusion injury, n (%)	48(13.7%)	215(34.6%)	<0.001***
**Antiplatelet plate drugs after 24 hours**			
Aspirin, n (%)	70 (19.9%)	122 (19.6%)	<0.001***
Clopidogrel, n (%)	27 (7.7%)	42 (6.8%)	
Aspirin+Clopidogrel, n (%)	216 (62.5%)	267 (42.9%)	
Others, n (%)	38 (10.9%)	191 (30.7%)	
Tirofiban used within 24 hours, n (%)	231 (65.8%)	358(57.6%)	0.011*
**Other events**			
Myocardial infarction, n (%)	11(3.1%)	28(4.5%)	0.296
Gastrointestinal bleeding, n (%)	5(1.4%)	23(3.7%)	0.042*
Epistaxis, n (%)	1(0.3%)	2(0.3%)	0.999
Infection, n (%)	143(40.7%)	383(61.6%)	<0.001***
**Laboratory test**			
ALT (U/L), median (IQR)	15.5 (9.7-25.0)	17.0 (9.5-27.0)	0.266
AST (U/L), median (IQR)	19.0 (13.8-26.0)	23.0 (14.0-32.0)	<0.001***
**Reperfusion after intervention (mTICI)**			
0-2a, n (%)	24(6.8%)	107(17.2%)	<0.001***
2b-3, n (%)	327(93.2%)	515(82.8%)	

Supplementary Table 2. The summary of evaluation metrics for each algorithm.

Algorithms	Accuracy [%]	Precision [%]	Recall [%]	F1	AUC
LGBM	73.77	73.16	75.18	74.09	0.824
LR	70.09	84.27	65.43	73.67	0.795

Supplementary Table 3. The effect of age on long-term outcome in EVT for AIS.

Age (y)	Favorable outcome in 90d mRS（n [%]）	Unfavorable outcome in 90d mRS（n [%]）	Total patients（n [%]）	OR (95%CI)	*P* (Value)
≤67	202 [51.5%]	190 [48.5%]	392 [40.3%]	1	-
> 67	149 [25.6 %]	432 [74.4%]	581 [59.7%]	3.08 (2.35-4.04)	<0.001***

Different age groups comparison (> 67 y group VS ≤67 group), ****P*<0.001.

Supplementary Table 4. The effect of NIHSS score on long-term outcome in EVT for AIS.

NIHSS Score	Favorable outcome in 90d mRS（n [%]）	Unfavorable outcome in 90d mRS（n [%]）	Total patients（n [%]）	OR (95%CI)	*P* (Value)
≤14	240 [52.7%]	215 [47.3%]	455 [46.8%]	1	-
> 14	111 [21.4%]	407 [78.6%]	518 [53.2%]	4.09（3.10-5.41）	<0.001***

Different NIHSS scores groups comparison (> 14 scores group VS ≤14 scores group), ***P<0.001.

Supplementary Table 5. Effect of different score segments in prediction scale on prognosis of EVT for AIS.

Prediction Scale	Favorable outcome in 90d mRS（n [%]）	Unfavorable outcome in 90d mRS（n [%]）	Total patients（n [%]）	OR (95%CI)	*P* (Value)
≤3	199 [62.0%]	122 [38.0%]	321 [34.0%]	1	-
> 3	152[23.31%]	500 [76.7%]	652 [67.0%]	5.36（4.19-6.88）	<0.001

Different prediction scale scores groups comparison (> 3 scores group VS ≤3 scores group), ***P<0.001.

Supplementary Figure 1. Distribution of 90d mRS of EVT for AIS in 169 patients in 2021.

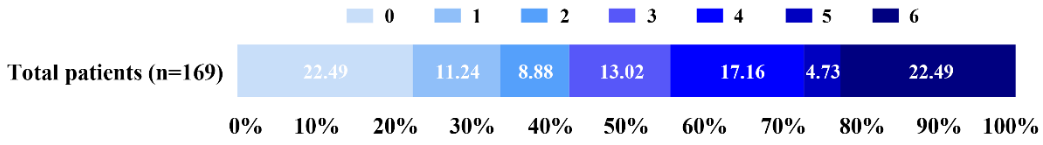

Supplementary Figure 2. ROC curve of the prediction scale for predicting outcomes after EVT for AIS in 973 patients from January 2018 to December 2020.

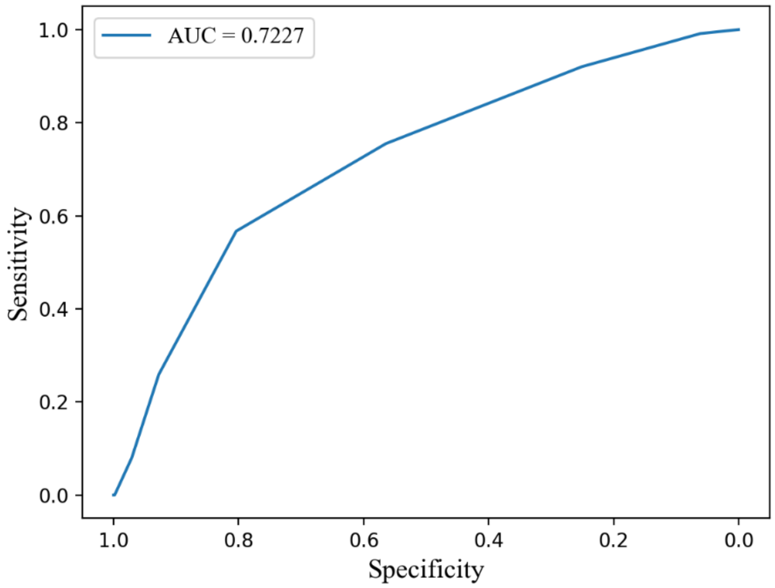

Supplement: Supplementary file 1 [file Presentation_1.pdf]
